# Surface sediment properties and heavy metal pollution assessment in the Pearl River Estuary, China

**DOI:** 10.1007/s11356-016-8003-4

**Published:** 2016-11-14

**Authors:** Guangming Zhao, Siyuan Ye, Hongming Yuan, Xigui Ding, Jin Wang

**Affiliations:** 1 Key Laboratory of Coastal Wetlands Biogeosciences, China Geologic Survey, Qingdao, 266071 People’s Republic of China; 2 Laboratory for Marine Geology, Qingdao National Laboratory for Marine Science and Technology, Qingdao, 266061 People’s Republic of China; 3 College of Marine Geo-science, Ocean University of China, Qingdao, 266100 People’s Republic of China

**Keywords:** Sediment properties, Heavy metals, Pollution assessment, Pearl River Estuary

## Abstract

Grain size and concentrations of heavy metals (arsenic (As), cadmium (Cd), chromium (Cr), copper (Cu), mercury (Hg), lead (Pb), and zinc (Zn)) of 148 surface sediments and activities of ^210^Pb and heavy metal concetrantions of one sediment core from the Pearl River Estuary were analyzed. The surface sediments were dominated by silt and sandy silt. Sediment type controlled the spatial distribution patterns of the heavy metals. The heavy metal concentrations in the sediments ranged from 3.34 to 37.11 mg/kg for As, 0.06 to 2.06 mg/kg for Cd, 12 to 130 mg/kg for Cr, 5.8 to 170.6 mg/kg for Cu, 0.01 to 0.25 mg/kg for Hg, 23 to 78 mg/kg for Pb, and 32 to 259 mg/kg for Zn. Both contents of clay and organic carbons were significantly positively correlated with heavy metals. The baseline values of elements in the study area were 12.97 mg/kg for As, 0.14 mg/kg for Cd, 68 mg/kg for Cr, 28.9 mg/kg for Cu, 0.08 mg/kg for Hg, 33 mg/kg for Pb, and 92 mg/kg for Zn. The metal enrichment factor (EF) and geoaccumulation index (Igeo) were calculated to assess anthropogenic contamination. Results showed slight to moderate Cd contamination in the region. Principle component analysis indicated that Cd could be attributed to anthropogenic sources; As and Hg were predominantly affected by human activities; and Pb, Cr, Cu, and Zn were associated with both natural and anthropogenic sources.

## Introduction

Heavy metals are of considerable environmental concern due to their toxicity, multiple sources, nonbiodegradable properties, and accumulative behaviors. Estuaries, which are regions of active land-ocean interaction, respond sensitively to natural processes and anthropogenic activities (Li et al. 2007). Estuarine sediments are recognized as an important sink for heavy metals and other contaminants (Ip et al. 2004) and have attracted much attention (Hu et al. 2013a; Venkatramanan et al. 2015; Wang et al. 2015; Wang et al. 2014b; Woods et al. 2012; Xu et al. 2014; Yang et al. 2015; Zhang et al. 2015a; Zhang et al. 2015b). Heavy metal contamination in sediments can affect water quality and thus the bioassimilation and bioaccumulation of metals in aquatic organisms, resulting in long-term implications for human and ecosystem health (Ip et al. 2007; Li et al. 2004; Raghunath et al. 1999). A thorough understanding of the depositional characteristics of surface sediments and pollutants is critical for the assessment of heavy metal pollution in marine environments (Xu et al. 2015b; Xu et al. 2015c).

The Pearl River Estuary (PRE) links the Pearl River, which is one of the largest rivers in southern China, and the South China Sea. The Pearl River is composed of three main river channels: West River (Xijiang) is the main channel in the network and is confluent with the East (Dongjiang) and North (Beijiang) Rivers in the lower reaches of the PRE (Lu et al. 2007). The lower alluvial and delta plains of the Pearl River are composed of Quaternary fluvial sediments (Lu et al. 2007; Zhang et al. 2007). In recent years, increasing population density and rapid industrial and agricultural development have resulted in severe stress on the aquatic environment of the PRE (Li and Huang 2008) and the introduction of many pollutants carrying heavy metals to the estuarine sediment (Chen et al. 2012). Although considerable effort has been made to investigate heavy metal pollution in PRE sediments (Chen et al. 2012; Ip et al. 2007; Wang et al. 2012; Yang et al. 2012; Ye et al. 2012; Yu et al. 2010), sampling density and heavy metal indexes are limited.

Based on high sampling density and seven heavy metal indexes, the present research aimed to (1) study the spatial distribution of grain size and heavy metals in surface sediments, (2) assess the state of heavy metal contamination using the enrichment factor (EF) and geo-accumulation index (Igeo), and (3) analyze and distinguish the possible sources of heavy metals.

## Materials and methods

### Sampling

**Fig. 1:**
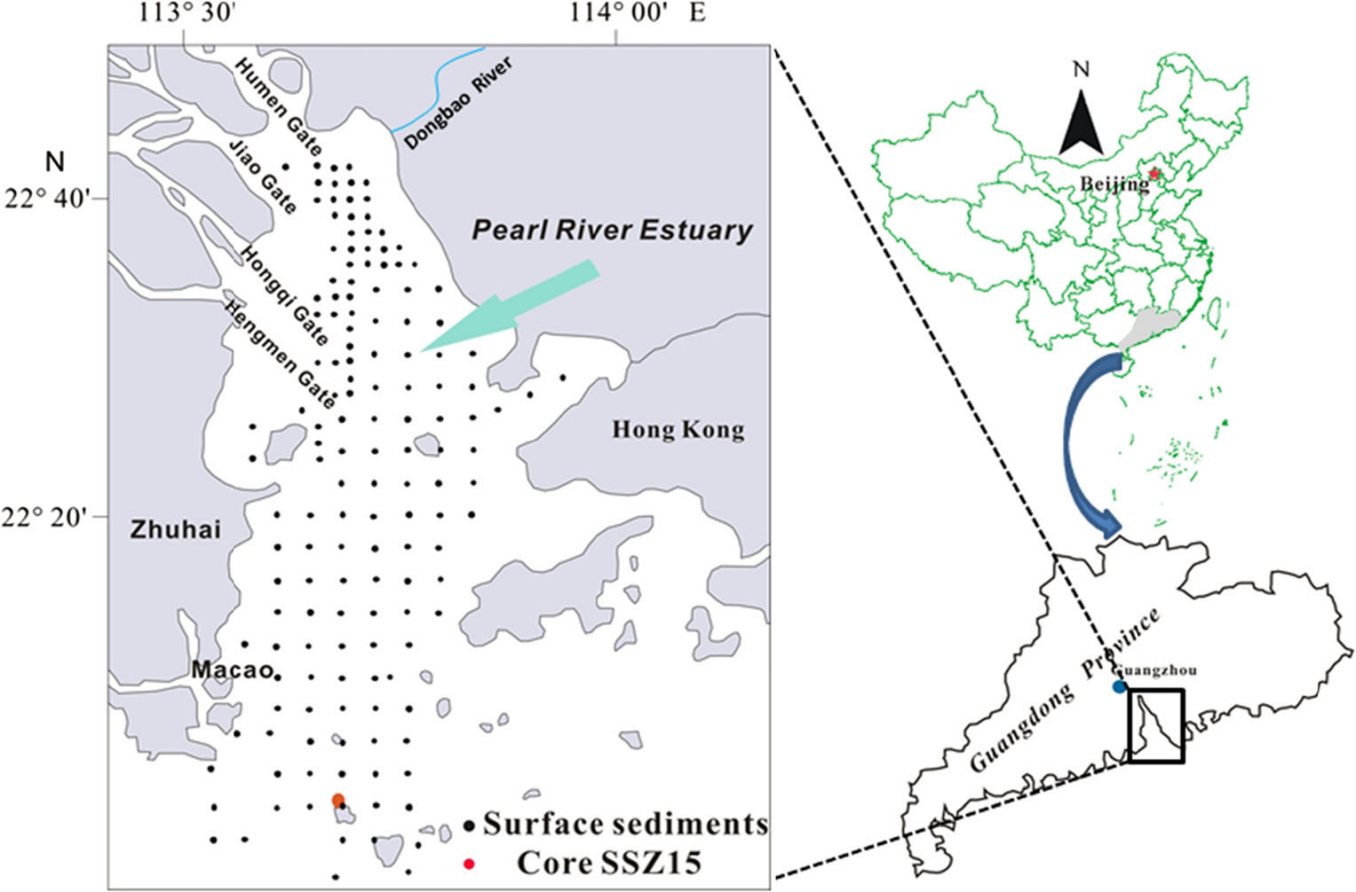
Location of the study area and sampling sites

We collected 148 surface sediment (0–5 cm) samples and one 196 cm long sediment core (SSZ15) from the PRE in January 2008 (Fig. 1). During sampling, each surface sample was placed in a clean cloth bag, and then enclosed in a polyethylene bag in the field. Once returned to the laboratory, the samples were fully air dried at room temperature, sieved through a 10 mesh (< 2 mm) nylon sieve, and then enclosed in a new polyethylene bag individually for later chemical analysis. The core sample was taken with a vibrating sampler and sliced in 4 cm long sections for grain size, element, and radionuclide analysis.

### Laboratory analysis

Sediment samples were pretreated with 10 % H_2_O_2_ to digest the organic matter. Excessive H_2_O_2_ solution was removed by heating and evaporation. After that, 0.5 % of sodium hexametaphosphate was added to the samples for sediment dispersal, with the mixture then analyzed with a Mastersizer 2000 laser particle-size analyzer (Malvern Ltd., UK) at the Experiment-Testing Center for Marine Geology, Ministry of Land and Resources, China (ISO 17025 laboratory accreditation). Grain-size parameters were calculated following classification from Folk and Ward (1957).

Samples were treated and determined according to the analytical elements. (1) Cd and Cu measurements were carried out using inductive coupled plasma mass spectrometry (ICP-MS). The samples were dried and ground to 63 μm before analysis, and the sediments were digested by adding a mixture of 9:5:2 HNO_3_ + HCl + HF to 0.5 g of the powdered samples and heating to 180 °C for 2 h (GB17378.5, Editorial Board of National Standards Press 1998) in closed Teflon bombs on a heating plate. These digestion steps were repeated with an additional acid until only a negligible amount of white residue remained. Each sample was then leached with diluted HNO_3_ and the solution was analyzed. (2) As and Hg were analyzed using atomic fluorescence spectrometry (AFS). (3) Cr, Pb, Zn, Al_2_O_3_, Fe_2_O_3_, K_2_O, CaO, Mn, and Sr were measured by wavelength dispersive X-ray fluorescence spectrometry (PANalytical AXIOS PW4400) after samples were pelletized, as per Xia et al. (2008). Calibration was made using certified reference materials and *α* correction was applied to correct for matrix interferences. (4) Organic carbon (Corg) was determined by wet oxidation in an acid dichromate solution, followed by back titration of the remaining dichromate using a ferrous ammonium sulfate solution. The analytical methods and detection limits of the above element determinations are listed in Table 1. Sediment reference materials (GBW07317, GSS1, GSS2 and GSS8) were used as analytical quality controls. The recoveries were between 90 and 99 % for all metals, with a precision of 10%.

**Table 1:** Analytical methods and detection limits

Indicator	Analytical method	Detection limit	Unit
As	AFS	1	μg/g
Cd	ICP-MS	0.02	μg/g
Cr	XRF	5	μg/g
Cu	ICP-MS	1	μg/g
Hg	AFS	0.003	μg/g
Pb	XRF	2	μg/g
Zn	XRF	2	μg/g
Mn	XRF	10	μg/g
Sr	XRF	5	μg/g
Al_2_O_3_	XRF	0.05	%
Fe_2_O_3_	XRF	0.05	%
K_2_O	XRF	0.05	%
CaO	XRF	0.05	%
Corg.	Electric potential	0.10	%

Both ^210^Pb and ^226^Ra were analyzed using the BE3830 gamma-ray spectrometer (Canberra Co., USA) at the Testing Center of the Qingdao Institute of Marine Geology, China Geological Survey, following a procedure similar to that of Xia et al. (2011). Counting uncertainties associated with sample measurements were typically less than 10%. Supported ^210^Pb activities were assumed to be equal to the measured ^226^Ra activities, and ^210^Pb activities (^210^Pb_xs_) were calculated by subtracting the supported ^210^Pb activities from total ^210^Pb activities (^210^Pb_tot_) (San Miguel et al. 2004). The sediment accumulation rates were calculated by the constant rate of ^210^Pb supply (CRS) model (Appleby and Oldfield 1992). The ^210^Pb geochronology was calculated using the equation:

1 $$ T={\lambda}^{-1}\cdotp \ln \left({A}_0/{A}_{\mathrm{h}}\right) $$

where *A*_0_ and *A*_h_ are the ^210^Pb_xs_ accumulation fluxes below the sediment-water interface and depth h, respectively, and *λ* is the ^210^Pb_xs_ radioactive decay constant (0.03114 year^−1^).

## Results and discussion

### Distribution patterns of grain size, Al, heavy metals, and Corg in sediments

**Fig. 2:**
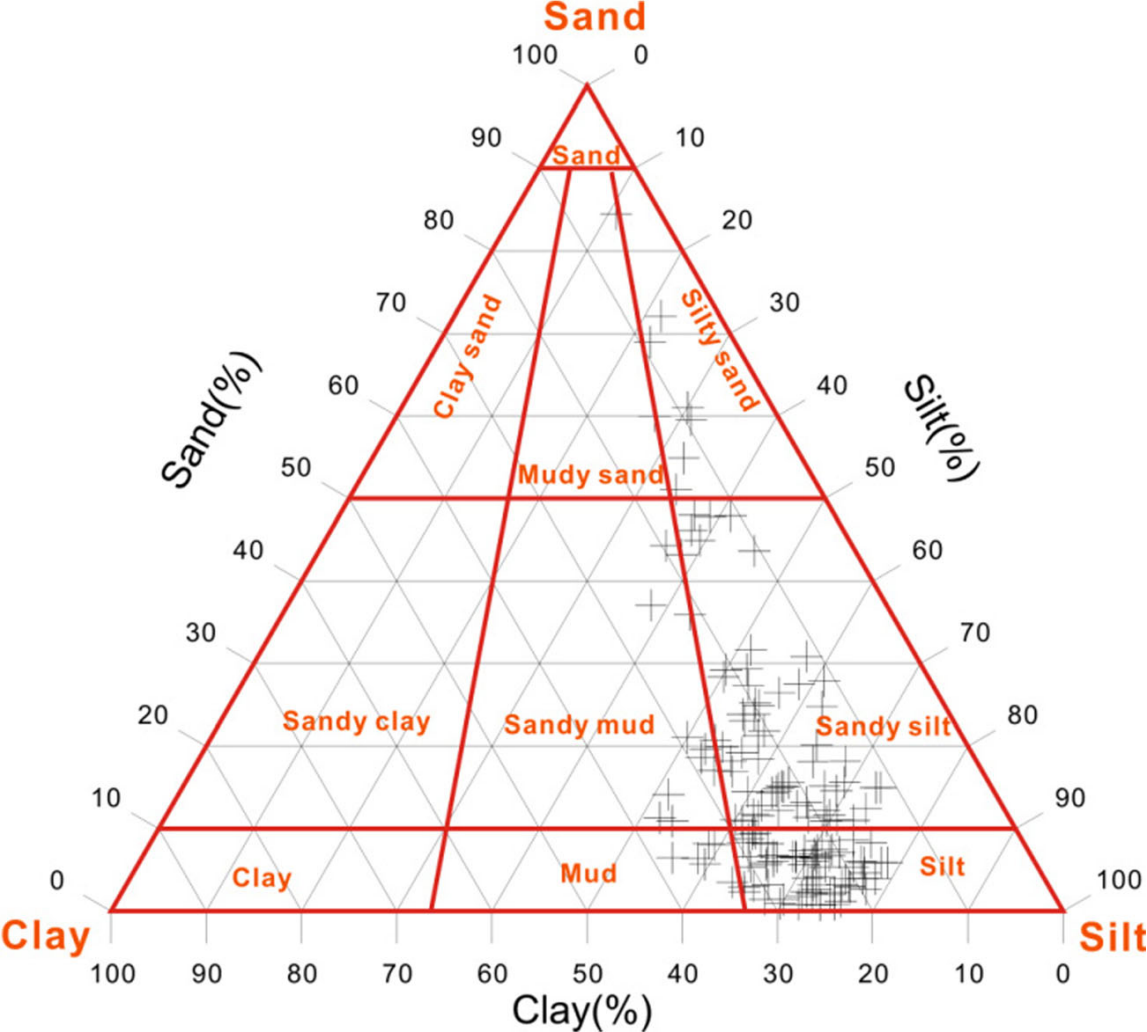
Percentage concentration of sand, silt, and clay (*black lines*) and sediment classification (*red lines*) in PRE

**Fig. 3:**
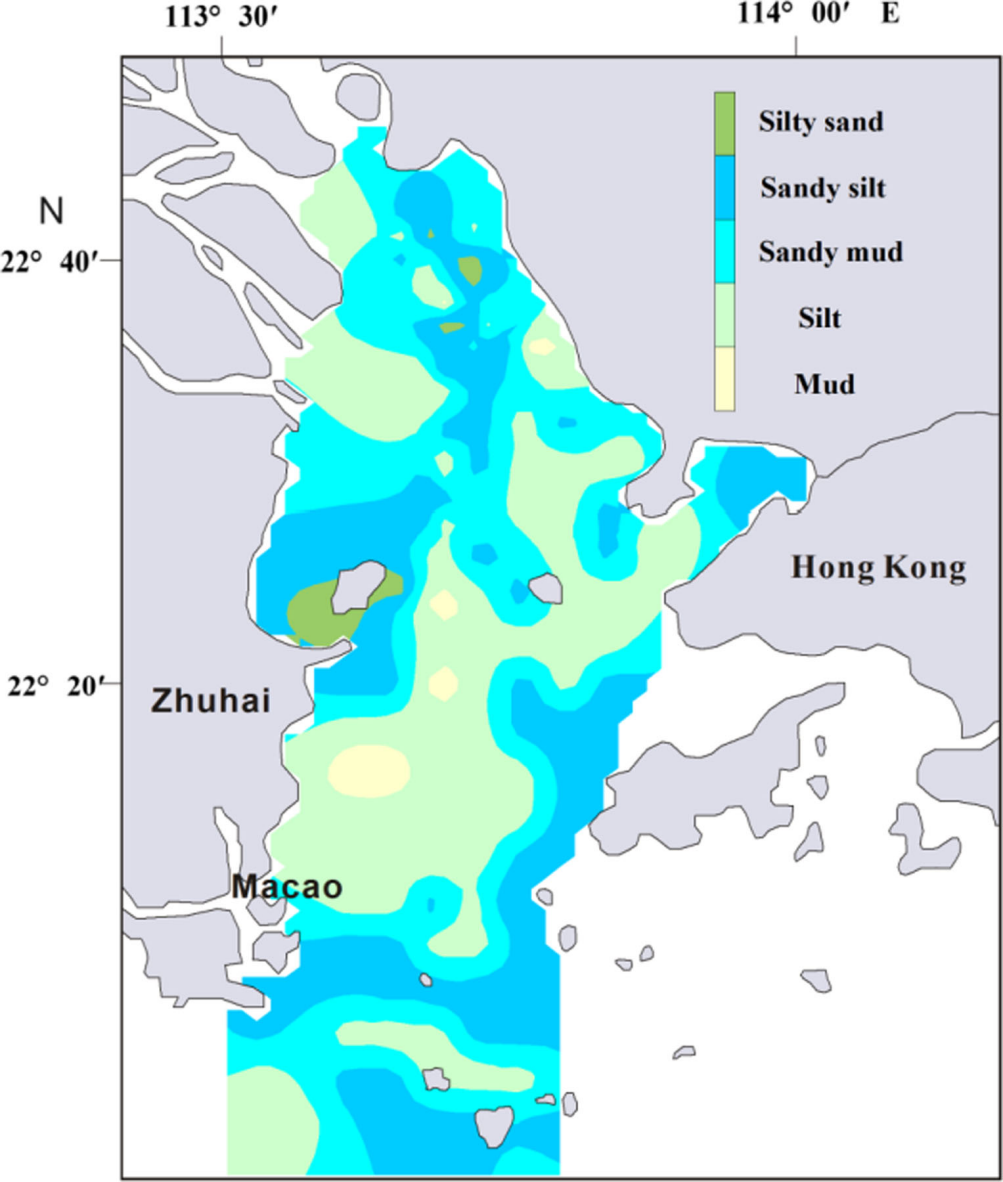
Spatial distribution of the surface sediment types in PRE

As shown in Fig. 2, the surface sediments in the PRE were dominated by silt, with a certain portion of clay and sand. On average, silt, clay, and sand accounted for 62, 22, and 16% of the material, respectively. Surface sediments mainly consisted of coarse-grained materials, indicating strong hydrodynamic conditions in the study area. According to Folk and Ward (1957), sediment in this region was classified into mud, silt, sandy mud, sandy silt, and silty sand (Fig. 2). Silt and sandy silt were the most widely distributed sediments, accounting for 84% of all samples (Fig. 3).

**Fig. 4:**
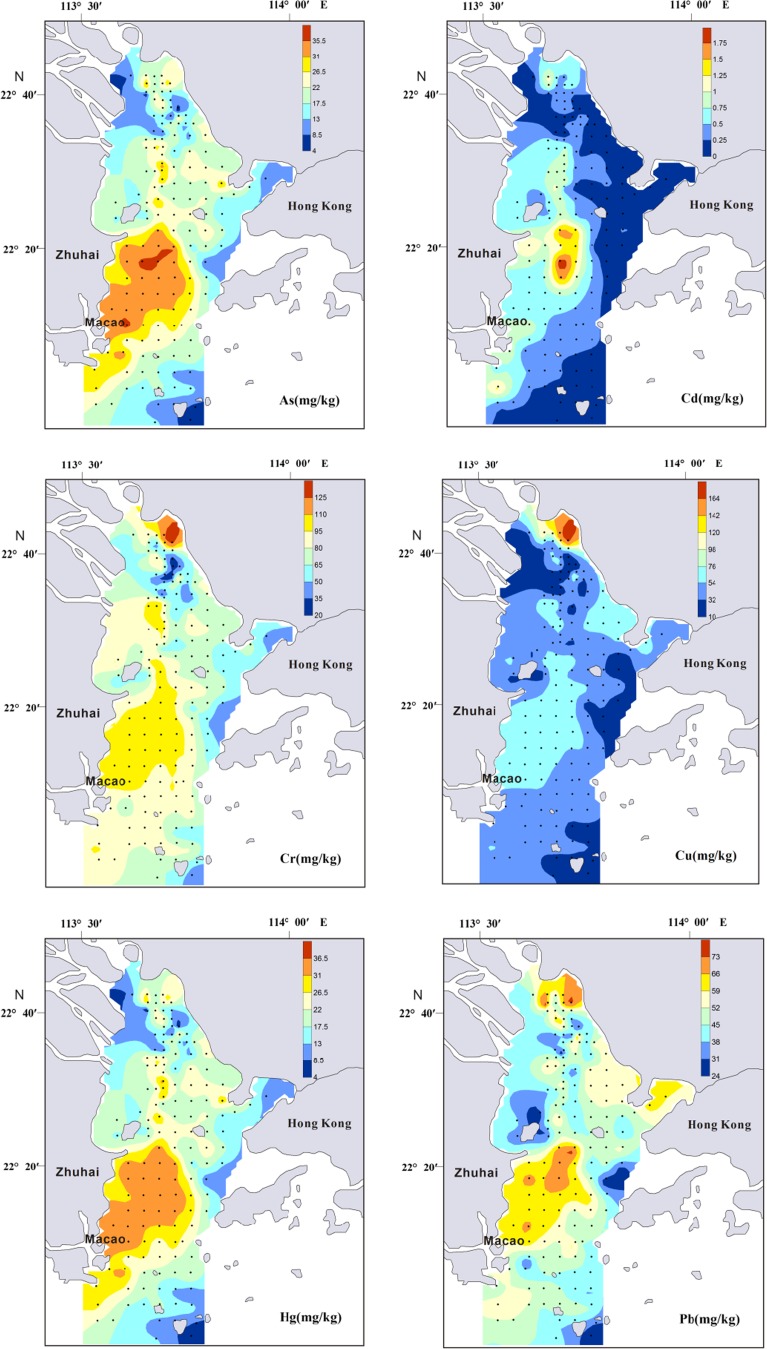

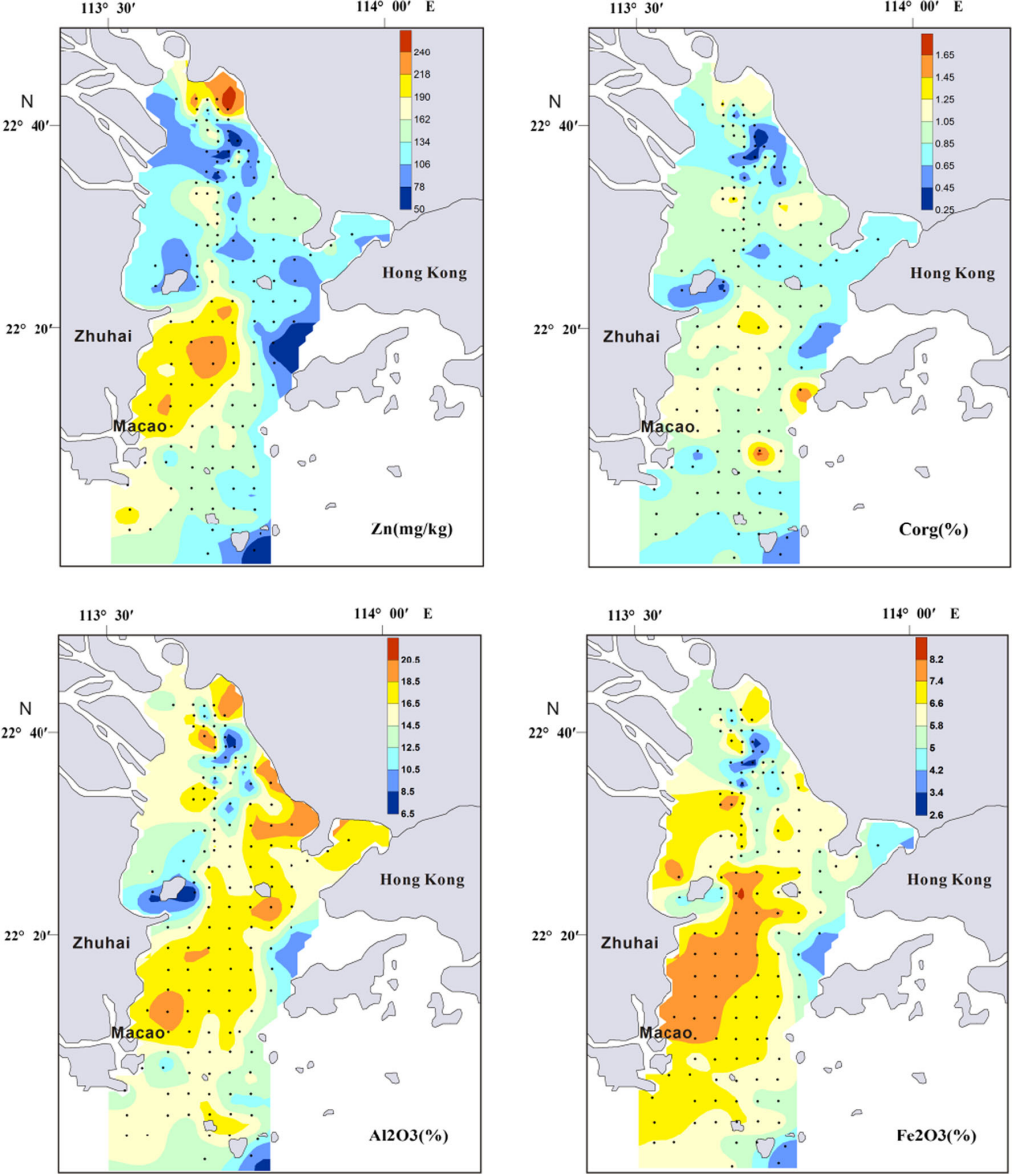
Concentrations of heavy metals, Corg, Al_2_O_3_, and Fe_2_O_3_ in the surface sediments of PRE

Overall, the distributions of surface sediment yielded a coarse-fine-coarse trend from north to south (Fig. 3). The northern region exhibited sandy deposition due to estuary runoff. Coarse particle distribution was basically parallel to the water channel and was also observed in the northeastern region of Zhuhai City. The middle region displayed transitional deposition, with a mixture of estuary and shelf sea silt. The southern region demonstrated sandy deposition based on its marine environment. Due to anthropogenic influence, especially nearby activities such as desilting and dredging, the original terrain of these regions, particularly that in the north, has been destroyed and depressions and shoals have increased, displaying no regular distribution. Hydrodynamic characteristics have changed, resulting in the diversity of grain size distribution in the PRE surface sediments (Xia 2005). Maximum enrichment of Al also occurred in the middle region but reduced both north and south. Minimum Al content was found around Qi-Ao Island, which is covered by silty sand (Fig. 4). Relative higher concentrations of Fe_2_0_3_ were recorded by sediments along the west-side of the PRE (Fig. 4).

The heavy metals (As, Cd, Cr, Cu, Hg, Pb, and Zn) and Corg of the surface sediment samples from the PRE are listed in Table 2. The concentrations of Corg ranged from 0.16 to 1.85%, with an average of 0.9%, which is higher than that of the Changhua River Estuary and adjacent shelf (0.55%) (Dou et al. 2013) and the Yangtze River Estuary (0.7 %) (Zhang et al. 2009a). The spatial distribution of Cd and Cu varied considerably, with the coefficients of variation of 79.18 and 48.67% (Table 2), respectively, demonstrating that these metals might be from point-source input and the similar finding was found in the previous study (Wang et al. 2014a). The spatial distributions of heavy metals and Corg concentrations in the study area are shown in Fig. 4 and, in general, showed similar patterns. Surface heavy metal distributions exhibit different underlying patterns due to different depositional environments, and distinct patterns of distribution have been identified in the PRE (Heise et al. 2010; Woods et al. 2012). Heise et al. (2010) utilizing principle component hierarchical cluster analysis to define and classify the PRE into four distinct areas: class 1, comprising of mainly coarser sediments; class 2, consisting of sediments dominated by eroded granite from the northeastern catchment area of the estuary; class 3, representing the influence of the marine environment and situated in the southernmost part of the outer Ling Ding Yang Estuary; and class 4, consisting of fine sediment deposited at high sedimentation rates and extending from the west to the Ling Ding Yang Channel. As shown in Fig. 4, all heavy metal concentrations decreased in the southeastern part of the estuary, an area with considerable marine influence (class 3), and the northern part of the estuary, an area with coarser sediments (class 1).

**Table 2:** Mean grain size, organic carbon (Corg), and heavy metal concentration in the surface sediments of PRE

Station	Mz (φ)	As (mg/kg)	Cd (mg/kg)	Cr (mg/kg)	Cu (mg/kg)	Hg (mg/kg)	Pb (mg/kg)	Zn (mg/kg)	Al_2_O_3_ (%)	Corg (%)
Min	2.32	3.34	0.06	12.00	5.80	0.01	23.00	32.00	5.65	0.16
Max	7.41	37.11	2.06	130.00	170.60	0.25	78.00	259.00	20.68	1.85
Average	6.29	21.99	0.46	78.37	46.76	0.13	49.66	143.10	15.24	0.90
S.D.	1.05	8.03	0.37	22.42	22.76	0.05	11.66	47.93	3.48	0.26
C.V.	16.63%	36.49%	79.18%	28.60%	48.67%	39.51%	23.48%	33.49%	22.83%	28.69%

*Mz* mean grain size, *Min* minimum values, *Average* average values, *S.D.* standard deviation, *C.V.* coefficient of variation

The concentrations of As, Cr, Hg, Pb, Zn, and Cd were high in the western shoal (class 3), consistent with that of other studies (Li et al. 2000a; Li et al. 2000b; Liu et al. 2003; Peng et al. 2003; Shi et al. 2006). A large number of terrigenous contaminants entering the estuary (Humen Gate, Jiaomen Gate, Hongqimen Gate, and Hengmen Gate) move southwest under the action of Coriolis force and coastal currents (Peng et al. 2003) and were thus deposited with sediments in the western shoal (class 3). In addition, mud and silt, which benefit the absorption and deposition of heavy metals (Qian et al. 1996; Zhang et al. 2009b), were widely distributed in the western shoal (Fig. 3). All heavy metals showed lower content in the eastern area (class 2) compared with that in the western shoal (class 3). High concentrations of Cr, Cu, and Zn accumulated on a small scale in the northeast of the PRE (Class 2) (Fig. 4), where the Dongbao River from Shenzhen city empties into the Pearl River (Jia et al. 2001). The sewage of the electroplate factory was the important source of Cr, Cu and Zn in Dongbao River (Jia et al. 2001). Thus, the sewage of the electroplate factory likely led to the increase in Cr, Cu, and Zn contamination in this area.

The Pearson correlation (PC) coefficients among the heavy metals, major elements, Corg, and clay in the sediments are shown in Table 3. All metals were positively correlated with clay content and Corg, suggesting that sediment size and organic carbon content might exert certain control over the abundance and regional distribution of heavy metals in the surface sediments of the PRE.

**Table 3:** Pearson correlation coefficient matrix of heavy metals, major elements, Corg, and clay in the surface sediments of PRE

	As	Cd	Cr	Cu	Hg	Pb	Zn	Corg	Al_2_O_3_	Clay	Fe_2_O_3_	Sr	CaO	K_2_O	Mn
As	1.000	0.794**	0.839**	0.814**	0.900**	0.780**	0.863**	0.722**	0.645**	0.408**	0.828**	−0.116	−0.253**	0.269**	0.730**
Cd		1.000	0.785**	0.756**	0.849**	0.604**	0.798**	0.629**	0.357**	0.361**	0.717**	−0.299**	−0.299**	−0.002	0.702**
Cr			1.000	0.855**	0.890**	0.746**	0.926**	0.821**	0.698**	0.462**	0.921**	0.011	−0.153	0.272**	0.750**
Cu				1.000	0.860**	0.834**	0.890**	0.770**	0.689**	0.468**	0.810**	−0.088	−0.278**	0.341**	0.647**
Hg					1.000	0.765**	0.923**	0.765**	0.572**	0.392**	0.844**	−0.055	−0.163*	0.149	0.749**
Pb						1.000	0.872**	0.723**	0.801**	0.499**	0.711**	0.011	−0.289**	0.588**	0.537**
Zn							1.000	0.813**	0.675**	0.426**	0.842**	−0.019	−0.211**	0.309**	0.723**
Corg								1.000	0.659**	0.418**	0.753**	0.034	−0.159	0.351**	0.593**
Al_2_O_3_									1.000	0.553**	0.734**	0.114	−0.227**	0.761**	0.470**
Clay										1.000	0.525**	0.055	−0.097	0.325**	0.415**
Fe_2_O_3_											1.000	0.116	−0.073	0.307**	0.799**
Sr												1.000	0.854**	0.064	0.013
CaO													1.000	−0.308**	−0.047
K_2_O														1.000	0.154
Mn															1.000

**Correlation is significant at the 0.01 level (two-tailed)

*Correlation is significant at the 0.05 level (two-tailed)

Comparisons of the heavy metals in the study area with those of other regions in China are listed in Table 4. Compared with that reported by Yu et al. (2010), the contents of Cr, Pb, and Zn in this study were lower, but Cu was comparable. Contrarily, the contents of As, Cd, Pb, and Zn were higher than those reported by Ye et al. (2012). Furthermore, the concentrations of heavy metals were much higher in the PRE than those reported in Daya Bay, Bohai Bay, and north Shandong Peninsula (Gao et al. 2010; Xu et al. 2015c; Yu et al. 2010). The contents of most heavy metals were higher than those in the South China Sea, Changjiang estuary, and eastern Beibu Bay but equal to that in western Xiamen Bay. Elevated high values of these heavy metals are probably due to the higher degree of industrialization in the Pearl River delta region and the increase in pollution due to rapid industrial development during the last three decades (Zhou et al. 2004). Primary sediment standard criteria are widely applied in environmental studies (CSBTS 2002). In comparison with these criteria, the contents of As and Cu in the current study were higher, whereas those of Cd, Cr, Hg, Pb, and Zn were close to the CSBTS. It is worth noting that the background value of Cu in the South China Sea is equal to that in the CSBTS (Zhang and Du 2005).

**Table 4:** Comparison of heavy metal of surface sediments in PRE(unit: mg/kg)

Location		As	Cd	Cr	Cu	Hg	Pb	Zn	Reference
Study area	Average	21.99	0.46	78.37	46.76	0.13	49.66	143.10	This study
Pearl River Estuary, China		na	na	106	45.7	na	57.9	176.8	Yu et al.(2010)
Pearl River Estuary, China		17.42	0.29	na	na	na	40.51	109.49	Ye et al.(2012)
Daya Bay, China		na	na	75.6	12.7	na	32.7	94.4	Yu et al.(2010)
Daya Bay, China		na	0.052	na	20.8	na	45.7	113	Gao et al.(2010)
Western Xiamen Bay,China		na	0.33	75	44	na	50	139	Zhang et al.(2007)
Eastern Beibu Bay, China		9.53	0.16	53.65	58.26	0.06	27.99	67.28	Dou et al.(2013)
Southern Bohai Bay, China		na	0.14	33.5	22.7	na	21.7	71.7	Hu et al.(2013c)
Liaodong Bay, China		8.3	na	46.4	19.4	0.04	31.8	71.7	Hu et al.(2013c)
Changjiang Estuary, China		na	0.26	78.9	30.7	na	31.8	94.3	Zhang et al.(2009a)
South China Sea, China		na	0.40	105	38.1	na	23.6	87.4	Zhu et al.(2011)
Near-shore area, north Shandong Peninsula, China		8.9	0.09	59	18.7	na	18.2	61	Xu et al.(2015c)
Primary standard, China		20	0.5	80	35	0.2	60	150	CSBTS(2002)

### Geochronology (^210^Pb) and background values

**Fig. 5:**
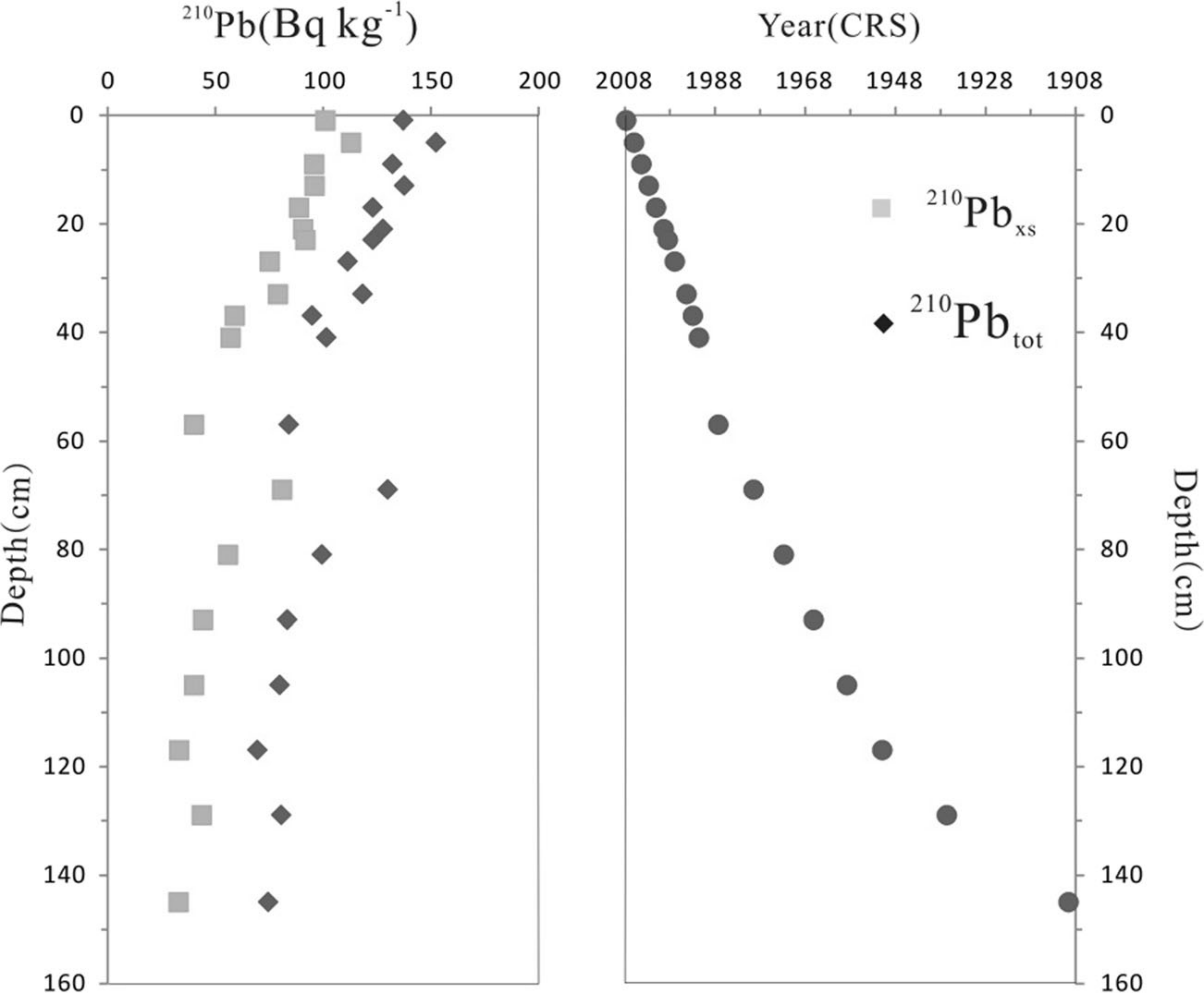
Profile distributions of ^210^Pb and ^210^Pb-derived chronology from the core SSZ15

The ^210^Pb depth profile is shown in Fig. 5. The average sedimentation rate of SSZ15 was 1.48 cm/a, which is in agreement with the results (0.5–1.5 cm/a) of other studies in this region (Chen 1992; Chen and Luo 1991; Ye et al. 2012). The elemental concentration in the Earth’s crust (Taylor and McLennan 1995) or abundance of upper crust shale (Rudnick and Gao 2003) is usually used as the baseline value for elements. However, the assessment of contamination levels when compared with a global standard (average shale or crust composition) is not always satisfactory due to the presence of local lithological anomalies (Zhou et al. 2014). Thus, the concentrations of sediments below 150 cm (about 100 years ago) in the core SSZ15 (12.97 mg kg^−1^ for As, 0.14 mg kg^−1^ for Cd, 68 mg kg^−1^ for Cr, 28.9 mg kg^−1^ for Cu, 0.08 mg kg^−1^ for Hg, 33 mg kg^−1^ for Pb, and 92 mg kg^−1^ for Zn) were selected as the background values in the study area.

### Assessment of heavy metal pollution

**Fig. 6:**
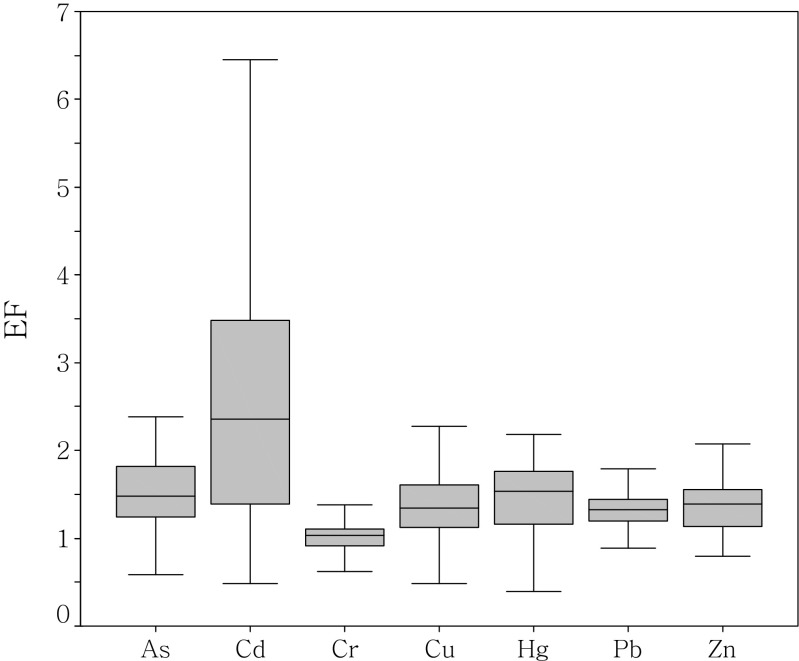
Enrichment factor (EF) of heavy metals in the surface sediments of PRE

The enrichment factor (EF) is widely used to discriminate between natural and anthropogenic sources and to reflect the status of environmental contamination. It is based on the use of a normalization element to alleviate the variations produced by heterogeneous sediments (Bastami et al. 2012; Christophoridis et al. 2009; Siddique et al. 2009; Xu et al. 2015a) and is calculated using the following equation:

2 $$ EF={\left({C}_n/X\right)}_{\mathrm{Sample}}/{\left({C}_n/X\right)}_{\mathrm{baseline}} $$

where *C*_*n*_ is the concentration of the considered element, and *X* is the normalization element. It was found that the relative proportion of a metal to Al in crustal material is fairly constant (Taylor 1964; Turekian and Wedepohl 1961). The ratio of heavy metals to Al can minimize the grain size effect between measured content and baseline values and reveal actual geochemical imbalance (Din 1992; Schropp et al. 1990). However, the use of a single element for normalization does not constrain which elements are enriched by human activities if used across the entire PRE, and it may be possible to better identify which elements are truly enriched by identifying and utilizing a normalization method appropriate to that region (Woods et al. 2012). Relative higher concentrations of Fe_2_0_3_ were found along the west-side of the study region (Fig. 4). In the case of the west side of the PRE, where Fe contents were elevated, the element Fe was therefore applied for normalization, while Al was used for the rest study areas. EF values between 0.5 and 1.5 (0.5 < EF < 1.5) indicate that the metals are entirely from crustal material or natural weathering processes, whereas EF values greater than 1.5 indicate an important proportion of noncrustal materials (e.g., anthropogenic influences) (Zhang and Liu 2002). The EF ranges of the heavy metals in the present study were as follows: As, 0.37–2.70 (average 1.49); Cd, 0.49–10.82 (average 2.9); Cr, 0.44–1.88 (average 1.01); Cu, 0.48–4.08 (average 1.39); Hg, 0.4–2.96 (average 1.45); Pb, 0.72–2.32 (average 1.36); and Zn, 0.79–2.64 (average 1.37). As shown in Fig. 6, the mean EF values of As and Hg were close to 1.5, suggesting a certain potential risk. The EF values of Cd (2.9) were more than 1.5 in all sampled areas, indicating significant Cd contamination in the study area.

**Fig. 7:**
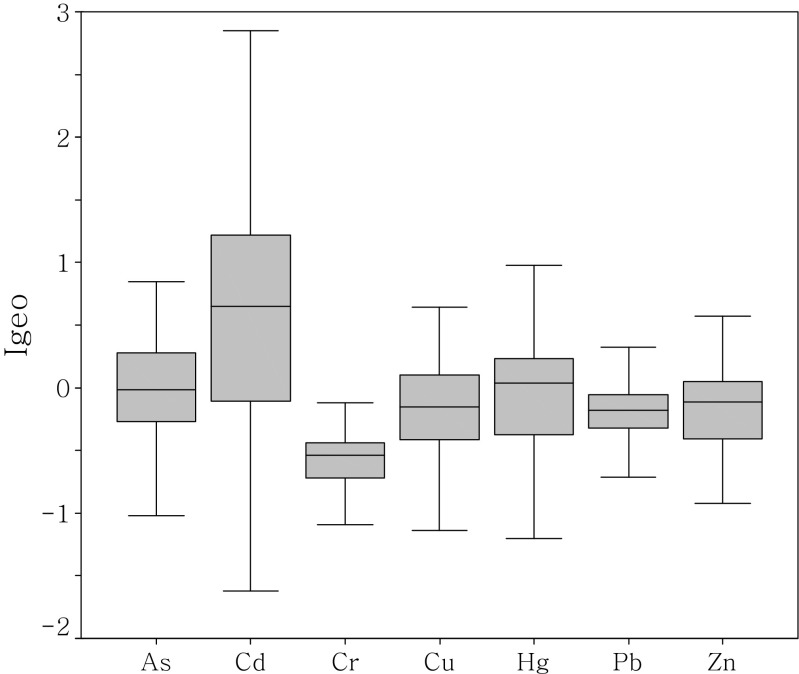
Geo-accumulation index (I_geo_) of heavy metals in the surface sediments of PRE

The Igeo, another commonly used criterion, was originally defined by Müller (1979) to evaluate heavy metal contamination in sediments by comparing current concentrations with pre-industrial levels and is defined by the following equation:

3 $$ {\mathrm{I}}_{geo}= \log {}_2\ \left[\left({C}_n/X\right)/\left(1.5\times {B}_{\mathrm{n}}\right)/X\right] $$

where *C*_*n*_ is the measured concentration of the examined metal (*n*) in the sediment, *B*_*n*_ is the background concentration of the metal (*n*), and factor 1.5 is the background matrix correction factor due to lithogenic effects. *X* is element for the normalization. Similar to the computations of EFs, elements Fe and Al were used separately in the western region and the rest of the regions as the normalization elements, The seven Igeo classes range from class 0 (Igeo ≤0) to class 6 (Igeo >5) (Müller 1981). The Igeo values of the heavy metals in this study are shown in Fig. 7 and ranged from −2.03 to 0.85 for As (average −0.08), −1.62 to 2.85 for Cd (average 0.63), −1.78 to 0.33 for Cr (average −0.59), −1.63 to 1.44 for Cu (average −0.20), −1.92 to 0.98 for Hg (average −0.12), −1.01 to 0.63 for Pb (average −0.17), and −0.92 to 0.81 for Zn (average −0.17). According to the Müller scale (Müller 1981), the Igeo values indicate no As, Cr, Cu, Hg, Pb, or Zn pollution in the study area as a whole, although some deviation was observed depending on the metal and sampling location. The Igeo value for Cd was between 0 and 1, generally indicating slight to moderate pollution in the PRE.

### Sources and transport of heavy metals

**Fig. 8:**
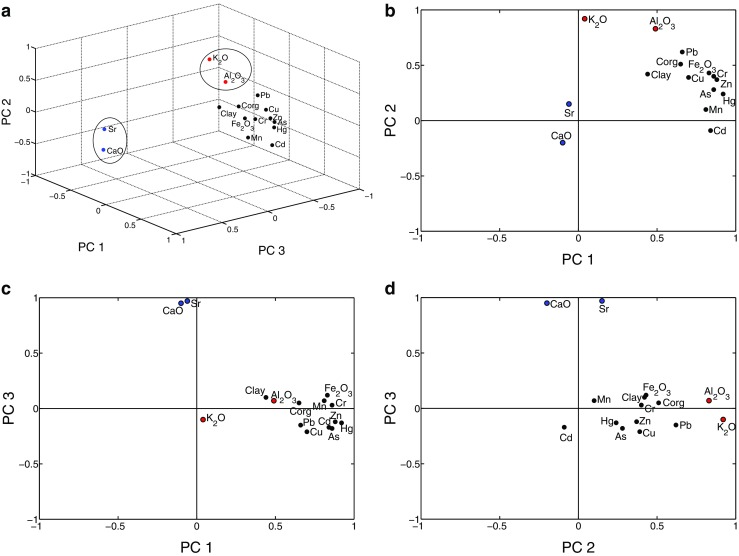
Principal component loading of heavy metals, major elements, Corg, and clay

Principle component analysis (PCA) is a common multivariate method used in environmental studies to investigate potential pollution sources (natural or anthropogenic) and their element characteristics (Han et al. 2006; Hu et al. 2013b; Li et al. 2013; Varol 2011). The rotated component matrixes of the PCA are presented in Table 5. The Kaiser-Meyer-Olkin (KMO) and Bartlett’s values were 0.853 and 3047.898 (df = 105, Sig < 0.01), suggesting that PCA might be useful in dimensionality reductions. The first three principal components accounted for 81.37% of total variance. The loading plot of the first three principal components of the surface samples is depicted in Fig. 8.

**Table 5:** Total variance explained and rotated component matrix of principal components analysis

Elements	PC1	PC2	PC3
As	0.856	0.277	−0.180
Cd	0.840	−0.085	−0.172
Cr	0.859	0.402	0.026
Cu	0.704	0.394	−0.213
Hg	0.924	0.241	−0.127
Pb	0.660	0.619	−0.149
Zn	0.876	0.373	−0.124
Corg.	0.654	0.509	0.46
Al_2_O_3_	0.488	0.830	0.69
Clay	0.440	0.421	0.100
Fe_2_O_3_	0.827	0.435	0.117
Sr	−0.055	0.146	0.965
CaO	−0.104	−0.195	0.954
K_2_O	0.038	0.920	−0.101
Mn	0.811	0.097	0.070
% of variance	46.30	21.41	13.66
% of cumulative	46.30	67.71	81.37

Extraction method: principal component analysis. Rotation method: varimax with Kaiser normalization. Rotation converged in three iterations

The first principal component (PC1), with high loadings of As, Cd, Hg, Cr, Cu, Zn, Pb, Fe_2_O_3_, Mn, and Corg, accounted for 46.30% of total variance (Table 5). Both EF and Igeo results indicated that Cd was clearly influenced by anthropogenic inputs, with As and Hg showing potential risk. Therefore, PC1 can be regarded as an “anthropogenic factor” mainly related to the discharge of industrial and agricultural wastewater and untreated urban sewage. Anthropogenic sources of heavy metals have been demonstrated in many estuaries worldwide (Diop et al. 2015; Jayaprakash et al. 2014; Pérez-López et al. 2011). In the PRE sediments, Cd, Cu, Pb, and Zn were significantly derived from anthropogenic sources (Li et al. 2001; Li et al. 2000b; Taylor and McLennan 1995; Zhou et al. 2004). This element group portrayed the anthropogenic input and accumulation in the estuarine sediments. According to studies on the chemical phase of metals in estuarine sediments, Fe/Mn oxides and organic/sulfide fractions are important geochemical phases for heavy metals in sediments, except for the dominant residual fraction (e.g., Cr, Hg, and Zn are mainly associated with the Fe-Mn oxide fraction; Cu and Pb are associated with the organic fraction) (Li et al. 2000a; Li et al. 2000b; Liu et al. 2003). In this study, Fe, Mn, and Corg showed high correlation with other elements in this group (Table 3), suggesting that heavy metals retained in the sediments bound preferentially to the Fe-Mn oxide fraction or organic matter.

The second principal component (PC2, 21.41% of total variance) had strong loadings on K_2_O and Al_2_O_3_ and moderate loadings for Corg, Fe_2_O_3_, clay, Pb, Cr, Zn, and Cu. Both Al and K are major constituents of common silicate minerals. Aluminum is extremely immobile in the marine environment and is usually held in a lattice of aluminosilicate minerals and regarded as a typical lithogenic element (Price et al. 1999). Therefore, Al and K_2_O mainly represent lithogenic origin from weathering and erosion of rocks and soil parent materials in the Pearl River catchment. Correspondingly, terrigenous sources of Al and K have also been observed in previous studies (Peng et al. 2003; Qi et al. 2010). In addition, the lithogenic elements (Al and K) are proportional to most anthropogenic elements, as expressed by their positive correlations in this study (Table 3). Moreover, PCA showed that Pb, Cr, Zn, Cu, Fe, and Corg had moderate loadings on PC2, indicating that these elements were derived, at least partially, from lithogenic sources bound in aluminosilicate minerals. Fine clay particles are an important carrier of trace metals to the coastal area (Ip et al. 2007; Yu et al. 2008).

The third principal component (PC3, 13.66% of total variance) demonstrated strong positive loadings for CaO and Sr, weak positive loading for Al and negative loading for K. Both Ca and Sr are essential components of marine biota and play an important role in the marine biogeochemical cycle. Furthermore, CaO and Sr are related to marine sedimentation processes and mainly originate from marine calcic biota (Barcellos et al. 1997; Rubio et al. 2000). The correlation coefficient between CaO and Sr reached 0.854 (Table 3), indicating that CaO and Sr probably originated from the same source. Similar findings in the PRE have been reported in other research (Zhou et al. 2004). Therefore, PC3 was characterized by a marine component.

## Conclusions

This study demonstrated that surface sediments in the study area were dominated by silt and sandy silt, indicating strong hydrodynamic conditions. The spatial distribution patterns of heavy metal concentrations were closely related to sediment type. Overall, Cd, Cr, Hg, Pb, and Zn in these sediments met the primary standard criteria of China (CSBTS 2002), though As and Cu did not. The heavy metal concentrations increased within the compositional range of other intertidal sediments in China. Both EF and Igeo showed slight to moderate Cd contamination in the PRE. Cd pollution, which was the most significant in the PRE, had negative loadings with PC2 and PC3, suggesting that Cd could be from anthropogenic sources. Both As and Hg appeared to mainly originate from human activity, whereas Pb, Cr, Cu, and Zn were supplied from both natural and anthropogenic sources.
